# Antiepileptic Effects of a Novel Non-invasive Neuromodulation Treatment in a Subject With Early-Onset Epileptic Encephalopathy: Case Report With 20 Sessions of HD-tDCS Intervention

**DOI:** 10.3389/fnins.2019.00547

**Published:** 2019-05-29

**Authors:** Oded Meiron, Rena Gale, Julia Namestnic, Odeya Bennet-Back, Nigel Gebodh, Zeinab Esmaeilpour, Vladislav Mandzhiyev, Marom Bikson

**Affiliations:** ^1^The Clinical Research Center for Brain Sciences, Herzog Medical Center, Jerusalem, Israel; ^2^Children Respiratory Unit, Herzog Medical Center, Jerusalem, Israel; ^3^Pediatric Neurology Department, Shaare Zedek Medical Center, Jerusalem, Israel; ^4^Department of Biomedical Engineering, The City College of the City University of New York, New York, NY, United States

**Keywords:** neonatal epileptic encephalopathy, seizure, high-definition transcranial direct current stimulation (HD-tDCS), electroencephalography (EEG), interictal epileptic discharges (IEDs)

## Abstract

The current clinical investigation examined high-definition transcranial direct current stimulation (HD-tDCS) as a focal, non-invasive, anti-epileptic treatment in a child with early-onset epileptic encephalopathy. We investigated the clinical impact of repetitive (20 daily sessions) cathode-centered 4 × 1 HD-tDCS (1 mA, 20 min, 4 mm ring radius) over the dominant seizure-generating cortical zone in a 40-month-old child suffering from a severe neonatal epileptic syndrome known as Ohtahara syndrome (OS). Seizures and epileptiform activity were monitored and quantified using video-EEG over multiple days of baseline, intervention, and post-intervention periods. Primary outcome measures were changes in seizure frequency and duration on the last day of intervention versus the last baseline day, preceding the intervention. In particular, we examined changes in tonic spasms, tonic-myoclonic seizures (TM-S), and myoclonic seizures from baseline to post-intervention. A trend in TM-S frequency was observed indicating a reduction of 73% in TM-S frequency, which was non-significant [*t*(4) = 2.05, *p* = 0.1], and denoted a clinically significant change. Myoclonic seizure (M-S) frequency was significantly reduced [*t*(4) = 3.83, *p* = 0.019] by 68.42%, compared to baseline, and indicated a significant clinical change as well. A 73% decrease in interictal epileptic discharges (IEDs) frequency was also observed immediately after the intervention period, compared to IED frequency at 3 days prior to intervention. Post-intervention seizure-related peak delta desynchronization was reduced by 57%. Our findings represent a case-specific significant clinical response, reduction in IED, and change in seizure-related delta activity following the application of HD-tDCS. The clinical outcomes, as noted in the current study, encourage the further investigation of this focal, non-invasive neuromodulation procedure in other severe electroclinical syndromes (e.g., West syndrome) and in larger pediatric populations diagnosed with early-onset epileptic encephalopathy.

**Clinical Trial Registration:** www.ClinicalTrials.gov, identifier NCT02960347, protocol ID: Meiron 2013-4.

## Introduction

In the current clinical investigation, the feasibility of high-definition transcranial direct current stimulation (HD-tDCS; Datta et al., 2009) as an antiepileptic treatment in a severe pediatric epileptic syndrome case was examined. Recent findings have highlighted the potential for tDCS to significantly alleviate epileptiform activity in children ages 6 to 15 (Yook et al., 2011; Auvichayapat et al., 2013, 2016). The stimulation electrodes are typically placed on the scalp with the cathode positioned over the epileptic focus and anode is placed elsewhere or extracephallicly, with the intention to reduce excitability (Ghai et al., 2000; Brunoni et al., 2012). To the best of our knowledge, conventional tDCS has never been evaluated for its potential to serve as a focal non-invasive anti-epileptic treatment in children with significant developmental delays suffering from early-onset epileptic encephalopathy and, other than our prior investigation, had not been examined in pediatric population younger than 4 years old (Meiron et al., 2018).

Unlike traditional tDCS (in a 1 × 1 configuration) used in prior antiepileptic interventions (Datta et al., 2009; Yook et al., 2011), randomized clinical trials (Auvichayapat et al., 2016), and sham-controlled double blind studies (San-Juan et al., 2017) examining the effects of tDCS on epileptic seizures and epileptiform activity in children and adults suffering from epileptic syndromes, HD-tDCS (in a 4 × 1 configuration) is expected to produce optimized neuromodulation by focally targeting specific paroxysmal seizure-related areas and producing specific current densities at the cortical level (Dmochowski et al., 2011; Kuo et al., 2013). The focality of HD-tDCS also minimizes neuromodulatory effects outside the target area (Edwards et al., 2013; Alam et al., 2016; Karvigh et al., 2017), as compared to conventional tDCS.

Newborns with OS frequently die during infancy, and survivors manifest psychomotor impairments, as well as continuous hypsarrhythmia accompanied with epileptic infantile-spasms (Watanabe et al., 1993a; Gaily et al., 2001; Ohtahara and Yamatogi, 2006; Beal et al., 2012). Ictal and interictal electroencephalography (EEG) can help monitor disease progression and the development of region-specific epileptic foci dominance. Specific pathological and epileptic EEG patterns such as changes in paroxysmal high-voltage slow wave delta peak-activity (i.e., maximum change in spectral power) within hypsarrhythmic electroclinical conditions can be observed over particular cortical regions in age-dependent epileptic encephalopathy cases such as West syndrome (WS) and OS (Gaily et al., 2001; Ohtahara and Yamatogi, 2006; Beal et al., 2012). In support, a recent fMRI-EEG study indicated that focal hypsarrhythmic epileptiform discharges [reflected by interictal epileptic discharges (IEDs) under certain scalp electrodes in infants suffering from hypsarrhythmia, infantile spasms, and developmental delay or WS] were significantly related to corresponding-focal hemodynamic changes, and most importantly, epileptiform paroxysmal-high-voltage changes in delta power were associated with BOLD signal changes in particular cortical and subcortical regions (Siniatchkin et al., 2007). Accordingly, paroxysmal high-voltage fluctuations in delta activity in these electroclinical conditions may be considered as secondary generalized epileptiform discharges. Slow-wave delta power was positively associated with BOLD signal only in the infants suffering from hypsarrhythmia and infantile spasms, as compared to an older group suffering from temporal lobe epilepsy and WS. This is an important clinical distinction since only patients with early onset hypsarrhythmia consistently showed significant correlations between EEG delta power and BOLD signals in the brainstem, thalamus, and as a group in putamen, all of which are neural substrates associated with infantile spasms with hypsarrhythmia, and OS etiology (Watanabe et al., 1993b; Ohtahara and Yamatogi, 2006; Siniatchkin et al., 2007). Furthermore, interictal spikes were associated with significant region-specific (e.g., the caudate nucleus) changes in BOLD signal in all infants suffering from hypsarrhythmia with infantile spasms.

Ten months prior to the current investigation (when the patient was 30 months old) we evaluated the safety and feasibility of HD-tDCS in reducing epileptiform activity and seizure frequency in a dose-escalation study (Meiron et al., 2018). We applied a dynamic montage approach allowing for the adaptation to the changing daily-epileptic-foci. Stimulation dose commenced with extremely low current intensities (0.1 mA) and was gradually increased to 1 mA over a period of 2 weeks. Our preliminary findings indicated a significant reduction in IEDs (e.g., reduced sharp wave amplitudes); however, significant alleviation of seizure frequency was not observed. All vital signs and physiological parameters were unchanged throughout our preliminary clinical trial and the intervention was considered safe without any adverse effects.

In order to investigate the inhibitory effects of 4 × 1 HD-tDCS on the most salient epileptiform activity in a 40-month-old child suffering from a severe neonatal epileptic syndrome known as Ohtahara syndrome (OS), we targeted the most dominant seizure-generating networks and collected video-EEG over the course of baseline (1 week), intervention (20 days with HD-tDCS), and post-intervention (32-days following the cessation of the HD-tDCS intervention) periods. Seizure frequency and IED parameters (e.g., interictal spike frequency and amplitudes) were considered the main clinical outcome measures in assessing changes from post-intervention period (32 days after termination of 20-day intervention) versus baseline period (1 week before onset of intervention). Averaged interictal delta power, and seizure-related paroxysmal changes in mean absolute delta power served as secondary clinical outcome measures indicative of secondary epileptiform activity associated with dysfunctional circuitry that may reflect the propagation of the most dominant epileptic focus activity (Siniatchkin et al., 2007). Specifically, primary clinical outcomes included the frequency of tonic spasms, myoclonic seizures, and tonic-myoclonic seizures (TM-S), and secondary clinical outcomes included paroxysmal changes in delta power topography, and IED parameters. Daily clinical monitoring (blood testing, online vital signs, respiratory rate monitoring) and registration was ongoing throughout the entirety of the clinical trial. In contrast to our initial dose escalation HD-tDCS intervention, the current clinical investigation utilized one 4 × 1 stimulation montage and current intensity of 1 mA over the course of the intervention (4 weeks). In light of functional MRI data revealing a close link between seizure-related BOLD signals and paroxysmal EEG delta power fluctuations (Siniatchkin et al., 2007) in infants with infantile spasms and hypsarrhythmia, and most particularly since tonic-myoclonic (TM) seizure onset is related to a significant decrement in relative delta power (Rosso et al., 2006), we hypothesized a reduction in mean seizure-related delta desynchronization, associated with alleviation of interictal spike activity during the post-intervention period versus baseline, as well as a reduction in TM seizure frequency and duration.

## Materials and Methods

The study was conducted at Herzog Medical Center Jerusalem, Israel. Approval was obtained from the Israel Ministry of Health and from the institute’s local institutional review board. The study was conducted in accordance with the Declaration of Helsinki, and written informed consent was obtained from the patient’s parents.

### Case

The patient was a 40-month-old child suffering from an early-onset epileptic syndrome called OS, which was the suspected diagnosis (based on his suppression-burst patterns and intractable seizure activity) at age 2 weeks. He was born after an uneventful, full term second pregnancy with birth weight of 3,160 g through a normal vaginal delivery, to healthy young parents with an older healthy child.

MRI scans and amino acid levels in the blood and CSF were within normal limits during the first 3 weeks after birth. Upon admission (at the Children Respiratory Unit, Herzog Medical Center, Jerusalem, Israel), at age 3 weeks, repeated seizures with bradycardic spells and oxygen desaturation episodes appeared and he was immediately ventilated through a tracheostomy, and fed through a PEG and has remained chronically ventilated and fed indefinitely. His current video-EEG displayed random asymmetrical high-voltage slow-wave spike activity referred to as hypsarrhythmia, and frequent interictal multifocal spikes with occasional irregular suppression burst (SB) patterns. Most of his ictal EEG activity was associated with intractable tonic spasms (most frequent), myoclonic seizures, and TM-S, which were the longest and most intense epileptic seizures. Seizure-related EEG activity seemed to be driven by paroxysmal changes in right hemisphere delta-frequency (1–2 Hz) spikes.

His routine antiepileptic medication included Clonazepam 1.5 mg/day, Vigabatrin 750 mg/day, and Topiramate 100 mg/day. His average seizure frequency was ranged from 50 to 100 epileptic seizures per hour. His overall EEG evolutionary changes (from age 3 months to 40 months) indicated a shift from regular SB patterns to modified hypsarrhythmia, with irregular periods of SB and slow-wave multifocal spikes. Although 75% of OS cases evolve to WS, intermittent irregular SB patterns consistently appeared in both sleeping and waking periods. This may support an OS diagnosis, which is extremely rare at this age-group, as most surviving cases after 6 months of age display hypsarrhythmia without SB patterns (Ohtahara and Yamatogi, 2006).

### Study Design

**FIGURE 1:**
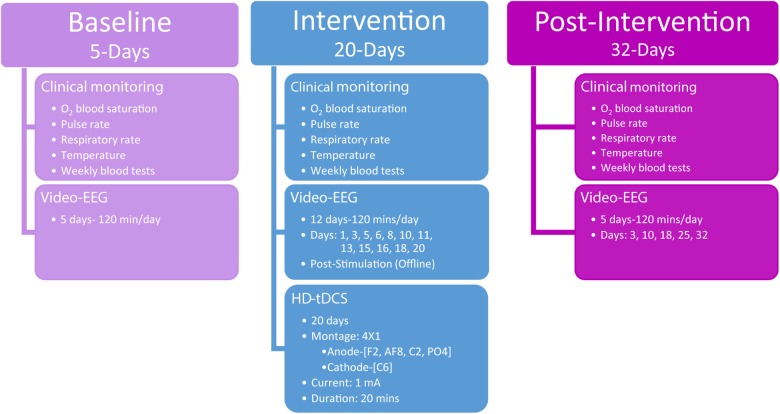
Study overview detailing the three phases of assessments including baseline, intervention, and post-intervention. The timing and procedures administered are detailed for each of the three phases.

The study consisted of a 5-day baseline-monitoring period prior to HD-tDCS intervention, a 20-day HD-tDCS intervention, and 32-days post-HD-tDCS intervention assessment period (Figure 1). The baseline-monitoring period consisted of five daily video-EEG recordings of 120 min per session (total of 600 min of baseline video-EEG), where epileptic discharge frequency and most dominant epileptic foci were monitored and quantified.

Following the baseline period, stimulation was applied according to the defined dose (see *HD-tDCS*) and was repeated over the course of 4 weeks (5-days per week). The 4 × 1 HD-tDCS montage was predetermined based on the scalp electrodes that indicated the largest paroxysmal amplitude and highest frequency of IED across frontal–temporal–parietal right-hemisphere locations, which was considered the most dominant seizure-generating neocortical area. During the intervention period, video-EEG data were acquired each day concurrently with HD-tDCS administration. For clinical monitoring purposes, intervention video-EEG data (120 min per session) were also acquired immediately after HD-tDCS sessions 12 times (120 min a day, at days 1, 3, 5, 6, 8, 10, 11, 13, 15, 16, 18, and 20; Figure 1) totaling 1,440 min of video-EEG indicating the child’s epileptiform activity immediately post HD-tDCS sessions during the intervention period.

Post HD-tDCS antiepileptic effects were assessed using video-EEG recordings across a 32-day post-intervention period (five 120-min sessions, totaling 600 min of post-intervention video-EEG) from post-intervention days 3, 10, 18, 25, and 32. As in the baseline period, post-intervention video-EEG analyses examined seizure frequency (number of seizure over a period of 120 min), IEDs parameters (spike/sharp waves), mean delta (0.5–3.5 Hz) power, and seizure-related mean maximal delta desynchronization across 7,200 s video-EEG segments per day.

Clinical monitoring (O_2_ blood saturation, pulse rate, respiratory rate, temperature, and weekly blood tests) was conducted across the entire clinical study and neurological assessments were administered at baseline (3 days prior to intervention) and immediately after the last HD-tDCS treatment session (day 20). Behavioral Pain Scale for critically ill patients (Payen et al., 2001) was administered before and after each HD-tDCS session to in order to record possible physical signs of discomfort or pain.

### Video-EEG Acquisition and Analysis

During the baseline and post-intervention period scalp EEG were acquired using a 32-channel shielded cap (WaveGuard cap, ANT Neuro, Netherlands) with online 50 Hz notch filter, band-pass 0.016–256 Hz, sampling rate 512 Hz, averaged reference, grounded at AFz, and amplified using an ANT 32 channel amplifier (ANT, Netherlands). Intervention scalp EEG data were acquired using a 32-channel shielded cap (WaveGuard cap, ANT Neuro, Netherlands) with 29 integrated HD holders (Soterix Medical Inc.). Electrode positions were based on the 10/10 international system. Data were sampled at 512 Hz with a 0.016–256 Hz bandpass filter and 50 Hz notch filter, referenced to CPz, grounded at AFz, and amplified using an eego sport amplifier (ANT Neuro, Netherlands) with a bandwidth of 0–520 Hz.

Offline data analysis (including automatic spike detection analysis) of baseline and pot-intervention EEG was performed using ASALAB (4.9.3; ANT Neuro, Netherlands), and MATLAB (R2015b; MathWorks, Natick, MA, United States). Offline, baseline and post-intervention EEG was cleaned using a zero-phase 0.25–70 Hz bandpass filter (filter steepness 24 dB/oct), and using the artifact-detection function within the advance source analysis software (ASA 4.9.3, ANT, Netherlands) for noisy amplitude changes (DC correction within the ±200 μV range). Fast Fourier transformation (FFT) with epoch-length 0.5 s across the entire 7,200 s (averaging 14,400 epochs) of clean EEG (with power spectra normalized) was employed in order to review the mean spectral-power-changes in delta (0.5–3.5 Hz), theta (3.5–7.5 Hz), alpha (7.5–12.5 Hz), and beta (12.5–30 Hz) bands and their scalp topography before and after HD-tDCS intervention. Spectral power density values were subjected to a log_10_ transformation for final statistical analysis. Seizure-related spectral analysis (noting spectral density power differences between 3 s pre-seizure time-window vs. 3 s post-seizure time-window, with 0.25 s offset) generating averaged spectral power head-maps of paroxysmal delta-band (0.5–3.5 Hz) activity across all visually marked ictal events, were collected and analyzed using Advanced Source Analysis software (ASA 4.9.3, ANT-Neuro, Netherlands). Seizure-related differences in SDP from pre to post-seizure-onset window were obtained by running an averaged FFT in 500 ms steps across 6,000 ms epochs of seizure-related events. We display the results only for the delta band where topography and power changes were most consistent with seizure-onset, and associated with paroxysmal changes in IED topography (Meiron et al., 2018). Supplementary Figure S1 provides an illustration of the raw EEG during a TM-S, and IEDs in an area representing the most dominant epileptic focus; under electrodes P4 and FC2.

The most dominant epileptic focus was predetermined during the baseline period according to the most salient topography in hypsarrhythmic slow-wave IED’s (Gaily et al., 2001), and most prominent topography of averaged seizure-related power changes in delta waves (Meiron et al., 2018). Accordingly, C6 electrode location, which was the midpoint between right parietal and right frontal most dominant epileptic foci (e.g., P4 and FC2showing the highest IED frequency, and mean spike amplitude) was chosen as the target location for placing the center-cathodal HD-tDCS electrode (Meiron et al., 2018). Those scalp EEG locations consistently displayed the most paroxysmal seizure-related delta and spike activity. The rationale behind the “midpoint” method for targeting the most dominant epileptic foci is that the HD-tDCS spatial configuration inhibits all these right hemisphere locations as they fall within the 4 × 1 HD-tDCS ring montage. Thus, although P4 and FC2 are hypothesized as most dominant foci, we cannot be sure that this epileptiform activity necessarily originates from only frontal or only parietal locations, therefore, it is likely the right motor cortex location that fall between these two IED locations (such as electrode C6), possibly also contributes to the generation of clinical seizures. All right hemisphere adjacent location represent seizure related dominant areas (Meiron et al., 2018), so we made sure that the cathodal stimulation covered all these proximal seizure-related locations. Thus, utilizing a large 4 × 1 ring configuration to cover all these areas is more likely to inhibit the right-hemisphere network that generates the IED’s and associated seizures.

In order to evaluate the changes in seizure frequency from baseline to post stimulation, seizures (and seizure classification) were recorded and noted by video-EEG and visually analyzed by pediatric epileptologist (i.e., child neurologist) and clinical electrophysiologist (authors OBB and OM, respectively). Epileptic tonic spasms were defined as a brief phasic contraction followed by a short tonic phase (Gaily et al., 2001) lasting around 1 s. Myoclonic seizures were defined as sudden muscle jerks, which lasted around 1–2 s. Tonic myoclonic seizures were defined as a brief tonic spasms followed immediately by myoclonic jerks last usually between 1.5–11 s. We inspected seizures of all durations (from 1 to 11 s duration), hypsarrhythmic interictal paroxysmal discharges (of random high-voltage slow waves and spikes of varying duration and location) were quantified according to Watanabe et al. (1993b) and Gaily et al. (2001) definitions of hypsarrhythmia observed in early onset electroclinical syndromes. IEDs were counted across 120 min EEG sessions using an automatic spike detection algorithm (ASA 4.9.3, ANT, Netherlands) calculating sharp waves (70–200 ms) and spike waves (0–70 ms) of negative polarity and an amplitude ratio five times larger than the averaged ongoing EEG activity. In addition to spike frequency, durations and peak amplitudes of spikes were extracted and noted by the spike-detection algorithm as well. These specific spike (sharp waves and spike wave) -parameters were previously supported as epileptiform markers associated with seizure frequency and topography in other cases of early onset epileptic encephalopathy (Watanabe et al., 1993b; Meiron et al., 2018). The spectral scalp maps for mean delta activity (2 Hz was used as it showed the maximal power peaks) were generated using MATLAB (version R2017b, with eeglab toolbox; Delorme and Makeig, 2004). The data were cleaned using automatic continuous rejection function and filter function employing a windowed sinc FIR filter algorithm with bandpass of 1–45 Hz (hamming window-type, PB dev = 0.002, SB att = -35 dB, transition bandwidth of 1 Hz). Fast Fourier Transform (FFT) was employed (Hanning window length: 512; FFT length: 512; overlap: 0) to extract the mean power of 2 Hz oscillations.

### HD-tDCS

**FIGURE 2:**
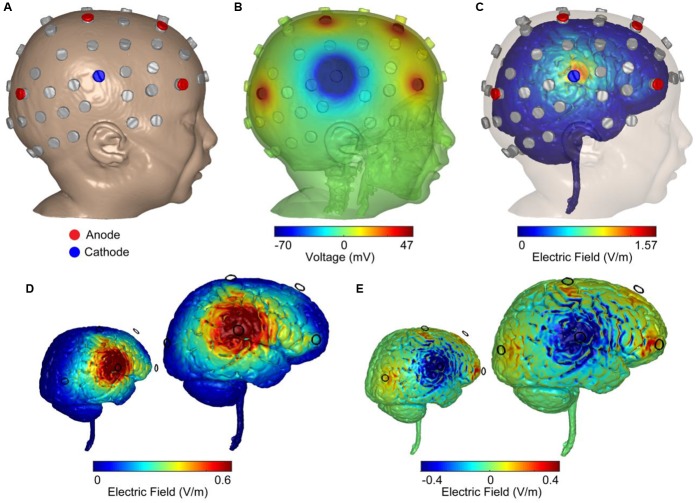
Computer simulations of 4 × 1 HD-tDCS intervention in an age-matched MRI-derived head model. **(A)** FEM MRI-derived model of an age-matched infant with electrode positioning is based on the 10/10 International system. Locations were used for recording EEG (gray) and stimulation (blue: cathode; red: anode). **(B)** Model-based prediction from FEM indicating scalp voltages across the head and at the anodes and cathode (false color: blue minimum = –70 mV, red maximum = 47 mV, green = 0 mV). **(C)** Model-based current flow field across the brain indicating maximal electric field under the cathode (false color: blue = 0 V/m, red = 1.57 V/m). **(D)** Finite-element model predictions of the normal electric field across the brain indicating electric field under the cathode (false color: blue = 0 V/m, red = 0.6 V/m). The color scheme maximized to 0.6 V/m (seen in adult brains under 2 mA of applied current) indicated a large right temporal lobe electric field. **(E)** Model-based radial electric field distribution across the brain indicating electric field under the cathode (false color: blue = –0.4 V/m- outward-radial electric field, inhibitory; red = 0.4 V/m-inward-radial electric field, excitatory).

Stimulation dose consisted of 1 mA of current delivered for 20 min a day, for 20 days, with a 4 × 1 HD-tDCS montage in an open-label design. The HD-tDCS center cathode was placed at the right central C6 location (C6 electrode location was the midpoint between most dominant frontal and parietal epileptic foci), and the four surrounding anodes were placed at anterior-frontal (AF8), frontal (F2), central (C2), and parietal–occipital (PO4) locations. While principled approached for EEG inversion are proposed (Cancelli et al., 2016; Dmochowski et al., 2017), the 4 × 1 montage is robust to simple configuration rules, producing significant current flow in the brain areas circumscribed by the ring-electrodes with a polarity based on the center electrodes (Alam et al., 2016). Therefore, the frontal anodes were 3 cm anterior (F2) and 8 cm anterior (AF8) to the dominant frontal focus which was under FC2 frontal location. The electrode PO4 anode was 3 cm posterior to the parietal dominant epileptic focus (P4), and the C2 anode was 6 cm dorsal to the parietal epileptic focus (Figure 2). The target area was predetermined based on the most salient scalp locations indicating maximal seizure-related delta power changes and paroxysmal interictal spike topography across a 5-day baseline period (Meiron et al., 2018). Stimulation was delivered through five Ag/AgCl sintered ring electrodes (4 mm radius), held in place by specially designed plastic casings and current was supplied using a Soterix Medical 1 × 1 LTE (extra voltage limited) stimulator and a 4 × 1 HD-tDCS adaptor (Hahn et al., 2013), while using established HD-tDCS methods (Villamar et al., 2013).

### HD-tDCS Computational Model

A high-resolution head model was generated based on an MRI of an age matched infant with 0.5 mm^3^ resolution in order to predict current flow patterns. A computational finite element model (FEM) of the head was employed to predict the spatial distribution of electric fields in cortex and the voltage distribution over the skin for safety considerations and stimulation efficacy. Automated segmentation was first performed using Statistical Parametric Mapping (SPM8) package (Wellcome Trust Centre for Neuroimaging, London, United Kingdom) in MATLAB in order to segment images into six different tissues with conductivities assigned to each: skin (0.465 S/m), skull (0.01 S/m), air (1 × 10^-15^ S/m), CSF (1.65 S/m), gray matter (0.276 S/m), white matter (0.126 S/m), electrode (5.8 × 107 S/m), and gel (1.4 S/m). Residual segmentation errors were corrected in ScanIP (Simpleware, Ltd., Exeter, United Kingdom) using a combination of segmentation tools. The resulting volumetric meshes were imported into a FEM solver (COMSOL, Burlington, MA, United States) for FE computation. The center cathode received 1 mA total current whereas the four anodes received symmetric 0.25 mA of current (Figure 2B). Maximizing the normal component of an electric field to 0.6 V/m, the approximate electric field observed in average adult brains under 2 mA of applied current, indicated a spread of electric field across the right temporal lobe (Figure 2D). Computing the magnitude of the radial electric field represents inward and outward components of the electric field (normal to the cortical surface; Figure 2E). An inward-radial electric field can be interpreted as excitatory/anodal stimulation whereas the outward-radial electric field could represent inhibitory/cathodal stimulation (Nitsche and Paulus, 2000; Bikson et al., 2004; Rahman et al., 2013).

### Statistical Analyses

Before examining statistically significant changes in outcome clinical variables, all seizure and epileptiform variables were subjected to normal distribution assessments using Kolmogorov–Smirnov tests. Variables that were normally distributed were subjected to parametric testing (paired sample *t*-tests), whereas non-normally distributed data were subjected to non-parametric statistical tests (Wilcoxon signed rank tests). In terms of interictal IED parameters, to avoid multiple comparisons, and because we were interested in the immediate effects following the intervention period, we compared only 1 day from post-intervention (immediately after 20 days of intervention) versus 1 day from baseline (3 days before HD-tDCS intervention). It is important to note that there were only five comparison points within baseline and within post-intervention for assessing changes in primary clinical outcome variables; thus, we acknowledge that assessing more days in future studies may further clarify the clinical benefits following a 20-day HD-tDCS intervention.

## Results

**FIGURE 3:**
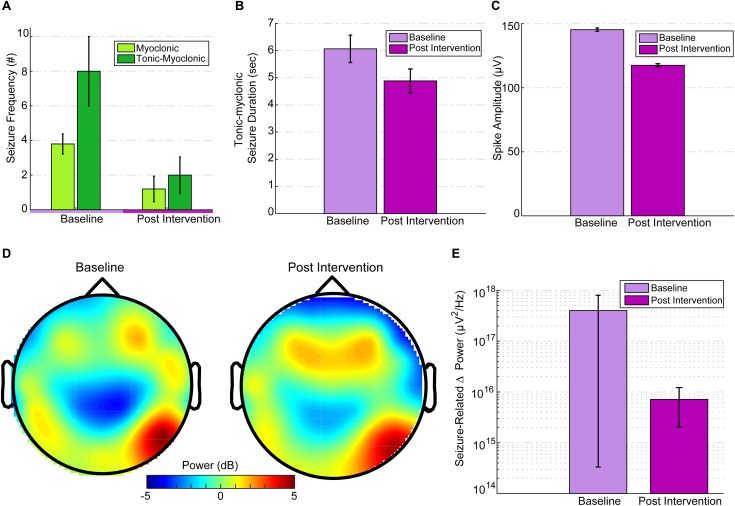
EEG seizure baseline and post-intervention comparisons. **(A)** Seizure frequency for baseline and post intervention divided into M-S and TM-S. M-S frequency was significantly reduced (*p* = 0.019) and TM-S were reduced by more than 50% post intervention. **(B)** TM-S duration between baseline and post-intervention periods. **(C)** Mean spike amplitudes at baseline (3 days before intervention) versus intervention day 20 (immediately after the last treatment session). Spike amplitudes were significantly reduced (*p* < 0.001) compared to baseline. Spike amplitudes over the post-intervention period were significantly reduced (*p* < 0.001) compared to baseline. **(D)** Topographic scalp maps of normalized delta activity (2 Hz) comparing baseline (3 days before stimulation) and post intervention (3 days after intervention). **(E)** There was a significant difference (*p* = 0.01) between seizure-related mean peak delta desynchronization between baseline and post intervention.

Tonic spasm frequency (TSF) was similar [*t*(4) = -0.29, *p* = 0.78] for both baseline (*M* = 189.8, *SD* = 68.65) and post-intervention (*M* = 225, *SD* = 214.54) periods. Post-intervention myoclonic seizure (M-S) frequency (mean: 1.2 ± 1.64) was significantly reduced [paired-samples *t*-test; *t*(4) = 3.83, *p* = 0.019] compared to baseline M-S frequency (mean: 3.8 ± 1.3; Figure 3A). Compared to baseline (*M* = 8, *SD* = 4.47), post-intervention (*M* = 2, *SD* = 2.34) TM-S frequency was not significantly [*t*(4) = 2.05, *p* = 0.1] lower. TM-S frequency was reduced by 72.88%, which constituted a clinically significant change, and represented an effective partial-response to the treatment defined as a seizure-frequency reduction >50% from baseline (Figure 3A). TSF, TM-S, and M-S frequencies across baseline and post-intervention periods were distributed normally. M-S (*M* = 3.8, *SD* = 2.16, range = 5) and TM-S frequency (*M* = 3.2, *SD* = 3.9, range = 7) during the intervention period (from days 5, 10, 13, 15, and 18) was similar to baseline M-S and TM-S frequency.

Since TM-S are of the longest durations (and seizure-intensity), a comparison between the TM-S durations from post-intervention (mean: 4.88 ± 1.45) versus the TM-S durations during baseline (mean: 6.06 ± 3.11) indicated that TM-S durations were non-significantly [*t*(24) = 1.46, *p* = 0.1] reduced by 19.47% at post-intervention versus baseline TM-S durations (Figure 3B). In order to avoid multiple comparisons, using a related samples Wilcoxon signed ranked test, we compared interictal spike frequency (summation of all spike waves; 0–70 ms, and sharp waves; 70–200 ms), spike peak amplitudes, and spike durations from intervention-day-20 (intervention day 20, immediately after the last HD-tDCS session) to the last baseline day (3 days before onset of intervention period) and found that the spike peak amplitudes were significantly reduced (*Z* = -45.8, *p <* 0.001) at post-intervention versus baseline (see Figure 3C). Spike durations were also significantly reduced at post-intervention versus baseline (*Z* = -27.49, *p <* 0.001). The interictal spike frequency 3 days prior to intervention was 10,876 versus 2,872 spikes at post-intervention day-20, indicating a 73.5% reduction in the frequency of IED. IED frequency during the baseline period (*M* = 8330.8, *SD* = 2017.86) was not significantly different [*t*(4) = 0.43, *p* = 0.68] from the IED frequency at post-intervention period (*M* = 7344.2, *SD* = 4018.57). The mean IED amplitudes were also not significantly changed [*t*(4) = -2.1, *p* = 0.1] at post-intervention (*M* = 154.54, *SD* = 13.69, range = 37.32) versus baseline (*M* = 142.26, *SD* = 6.03, range = 16.04).

Mean absolute spectral power of whole-brain (from 32 electrode location) delta frequency (Fast-Fourier transformed and averaged across 14,400 epochs of 0.5 s) across the 2-h baseline session (3 days before intervention) was lateralized asymmetrically and most dominant at right parietal-temporal (and slightly right-frontal) locations (see Figure 3D, illustrating normalized 2 Hz changes from baseline to intervention) and subdominant at contralateral temporal T7 electrode location. Post-intervention (3 days after intervention) mean whole-brain relative change in delta (0.5–3.5 Hz) power was 45.5% lower than baseline. To avoid multiple comparisons, we considered the change in mean delta power only between these two specific days before and after the intervention. This comparison reflected time-points that were closest to the onset and termination of the intervention. The mean relative change in delta power at post-intervention was reduced particularly at target electrode locations (P8-C6 and T8-F8 at right hemisphere) under the HD-tDCS 4 × 1 target-area (most dominant baseline epileptic focus), and the reduction was more pronounced over-the target-right hemisphere (Figure 3D).

Seizure-related delta-desynchronization (delta ERD) during post-intervention was significantly lower (*Z* = 337, *p* = 0.01) than delta-ERD at baseline (Figure 3E). All seizure-related (during tonic-myoclonic, tonic spasms, and myoclonic seizures only) paroxysmal delta ERD peaks (at least 500% change-in induced delta power at a 4 s interval following seizure onset relative to -0.1 to -0.3 s pre-seizure interval) were found mainly within the lower delta range (0.5 Hz–2 Hz). Across the entire baseline period (600 min of EEG) 68 delta ERD peaks were detected versus 29 delta-ERD peaks at post-intervention period (600 min of EEG), indicating a 57% reduction in ictal delta ERD activity after 20 days of HD-tDCS treatment. Therefore, not all the observed seizures were accompanied by 500% change in peak delta power and were excluded from the final peak delta ERD analysis.

The baseline neurological examination indicated severe axial hypotonia, and the child did not show any sign of communication. He responded to pain stimulation with non-specific limb movement. He was unable to make eye contact or follow an object visually and did not respond to acoustic stimuli. There were only occasional spontaneous limb movements. Contractures with limited range of motion of fingers, wrists, ankles and knees were noted bilaterally. Deep tendon reflexes were absent. There was no clonus. Plantar responses were extensor bilaterally. Face was symmetric with minimal movements and expressions. There was no titubation or dysmetria. Gag reflex was weak and corneal reflex elicited on the right but not on the left eye. At post-intervention examination there was no change in neurological status except this time the corneal reflex was elicited bilaterally.

**FIGURE 4:**
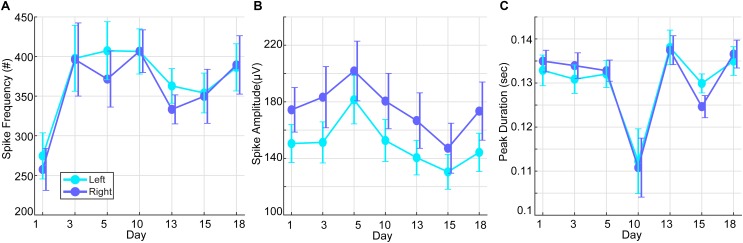
Interictal epileptic discharges (spike waves and sharp waves) during the intervention period. **(A)** Mean spike frequency of the right and left hemisphere over days of stimulation (baseline prior to HD-tDCS: *M* = 8330.8, *SD* = 2017.86). **(B)** Mean spike amplitudes of the right and left hemisphere over days of stimulation (baseline prior to HD-tDCS: *M* = 142.26, *SD* = 6.03 μV). **(C)** Mean spike duration of the right and left hemisphere over days of stimulation (baseline prior to HD-tDCS: *M* = 0.093, *SD* = 0. 0044 s).

Over the course of the HD-tDCS intervention period, IED parameters were monitored to verify a consistent decline in paroxysmal epileptiform activity. IED frequency and peak amplitudes showed the largest reduction after intervention days 1, 13, and 15, while spike durations showed the most pronounced reduction at day 10 of the intervention (Figure 4). No significant differences were observed between the left and right hemisphere spikes, and the IED parameters were not significantly changed within the intervention period. To observe the acute IED dynamics during the treatment intervention, treatment days 1–18 were compared. Note that mean spike frequency during the intervention period showed a pronounced decrease from baseline days (*M* = 8330.8, *SD* = 2017.86). To examine the relationship between the epileptiform activity on intervention-day 20 with post-intervention days, we examined the correlations among these measures’ peak amplitudes. IED peak amplitudes on intervention-day 20 of the intervention were significantly related (all were subject to Spearman’s Rho test; -0.142 ≤*R* ≤-0.295, *p* < 0.001) to IED peak amplitudes on all post-intervention days (3, 10, 18, 25, and 32 days post intervention).

## Discussion

The efficacy of tDCS to suppress epileptiform activity and epileptic seizures was demonstrated in animal models (Nitsche and Paulus, 2009) and patient populations with focal epilepsy due to abnormal cortical development (Fregni et al., 2006). In patient populations, 20 min of cathodal tDCS positioned at their epileptic focus significantly reduced epileptic discharges, but had only a trend-wise effect on seizure frequency (Fregni et al., 2006). There is a broad consensus across translational animal studies and clinical trials on the putative mechanisms of direct current stimulation (Sunderam et al., 2010). tDCS will produce membrane polarization with current directed in the cathodal direction producing somatic hyperpolarization (Chan and Nicholson, 1986; Bikson et al., 2004) which reduces neuronal excitability (Gluckman et al., 1996; Bikson et al., 2004). When tDCS is sustained for minutes, it can produce lasting change in excitability (Bindman et al., 1964; Nitsche and Paulus, 2000). tDCS is among a constellation of emerging and investigational brain stimulation techniques (both invasive and non-invasive) for epilepsy (Wu and Sharan, 2013; Nune et al., 2015; Eastin and Lopez-Gonzalez, 2017; Kwon et al., 2018), but is unique in acting through direct hyperpolarizing rather than decreasing excitably secondary to neuronal activity (e.g., LTD, desynchronization).

In this case, our results suggest an effective, and significant clinical response, as well as a significant reduction in IED amplitudes immediately after the last day of intervention, via application of a focal, non-invasive neuromodulation procedure. The reduction in IED amplitudes was not sustained during the post-intervention period, while the reduction in myoclonic seizures was noted and sustained during the post-intervention period. In addition, it is important to note that although targeted dominant foci seem to receive less inhibitory field than its proximal C6 motor-network center-cathode location (see Figure 2E), these dominant foci locations were applied as the topographical boundaries of the seizure-generating zone, targeting the area under the HD-tDCS right-hemisphere ring configuration between the frontal and parietal foci locations. Thus, the maximal inhibitory current at the common adjacent motor-network residing between frontal–parietal dominant foci most likely generated secondary inhibitory effects at frontal–parietal dominant foci locations. Specifically, we utilized HD-tDCS to treat a 40-month-old child suffering from a severe electro-clinical syndrome (i.e., OS, organic etiology associated with brain-dysgenesis); a condition that ultimately results in severe developmental retardation, high frequency of daily seizures, and early death at infancy (Ohtahara and Yamatogi, 2006).

Importantly, there are only a few sporadic early-onset epileptic encephalopathy cases that display a sufficient response (e.g., seizure control) to conventional anti-epileptic treatments (e.g., ACTH, clonazepam, valproate, vigabatrin or ketogenic diet). Although the current investigation is limited by one case, our findings support a significant alleviation of myoclonic seizures and attenuation of epileptiform-hypsarrhythmic features that are related to electroclinical-syndrome severity and prognosis (Siniatchkin et al., 2007). Seizure-related delta reactivity during the post-intervention period and IED amplitude/durations on intervention day 20 were significantly reduced, which was clinically cross-validated by a significant reduction in myoclonic seizure frequency, and a 72% reduction in TM-S. TSF was not significantly reduced, however, it’s underlying paroxysmal (high-voltage) delta activity, associated with increased brain-stem BOLD signal (Siniatchkin et al., 2007), was reduced by 57%. The observed clinical response (attenuation of M-S and TM-S frequency) was supported by significantly lower seizure-related whole-brain delta ERD at post-intervention (post-intervention days 3 to 32). More so, the IED amplitudes on intervention day 20 were significantly associated with IED amplitudes within the five different post-intervention days, possibly implying a common source of epileptiform activity. The reduction in epileptiform activity observed on intervention day 20 may have contributed to the observed clinical benefits past the active intervention period, such as the reduction in myoclonic seizures observed during the post-intervention period. The reduction in slow-wave activity was observed over the targeted seizure-generating network, indicating lower hypsarrhythmic delta activity at right parietal–temporal electrode locations, which showed the most paroxysmal IED’s at baseline. This localized reduction attested to the focal effects of HD-tDCS over the targeted cortical region.

HD-tDCS can be guided to stimulate any brain regions, including deep structures, and multiple foci (Dmochowski et al., 2017; Huang et al., 2018). Ongoing studies may inform which cortical or brain-stem substructures (Siniatchkin et al., 2007) may be targeted using customized HD-tDCS montages to reduce specific epileptiform activity patterns related to tonic-spasm generation in pediatric cases suffering from early-onset epileptic encephalopathy syndromes. This case study supports ongoing HD-tDCS seizure-control trials in pediatric epilepsy including other severe electroclinical syndromes (displaying hypsarrhythmia and intractable seizures) such as early myoclonic encephalopathy, Lennox-Gastaut syndrome, and WS, and cross-validated in epileptic patients with childhood focal epilepsy.

## Ethics Statement

This study was carried out in accordance with the recommendations of Herzog Medical Center Institutional review board with written informed consent from the legal guardian (parents). The subject’s parents gave their informed consent in accordance with the Declaration of Helsinki. The protocol was approved by the Israel Ministry of Health and the Herzog Medical Center’s Institutional Review Board.

## Author Contributions

All co-authors contributed significantly to the execution and supervision of the current study. OM was responsible for data collection, data analysis, interpretation of findings, overseeing the execution of the study, and the composition of the manuscript. RG was responsible for data collection, supervision, and composition of the manuscript. JN was responsible for data collection, supervision, and clinical evaluations. MB was responsible for data analysis, supervision, and manuscript composition. NG was responsible for data analysis and composition of the manuscript. OB-B was responsible for data collection including clinical evaluation and manuscript composition. ZE was responsible for data analysis. VM was responsible for data organization and data analysis.

## Conflict of Interest Statement

The City University of New York has patent on brain stimulation with MB as inventor. MB has equity in Soterix Medical Inc. which makes brain stimulation devices. The remaining authors declare that the research was conducted in the absence of any commercial or financial relationships that could be construed as a potential conflict of interest.

## Supplementary Material

FIGURE S1 Raw EEG activity during tonic-myoclonic seizure. Red vertical lines represent the onset and offset of the seizure, orange horizontal line represents the duration of the seizure (close to 4 seconds). The left purple line represent interictal epileptic discharges on several channels (e.g., P8, C4, CP2) at approximately one second before the seizure onset. The light-blue vertical lines mark specific channels that display IED onset before (at P4 electrode) and after (at FC2 electrode) the seizure. The entire figure represents 10 seconds of EEG, each second indicated by gray vertical lines.
