# Supplementary figures and images for: Antiepileptic Effects of a Novel Non-invasive Neuromodulation Treatment in a Subject With Early-Onset Epileptic Encephalopathy: Case Report With 20 Sessions of HD-tDCS Intervention
Source: Front Neurosci. 2019 May 29;13:547. doi: 10.3389/fnins.2019.00547 (PMC6548848; doi:10.3389/fnins.2019.00547)

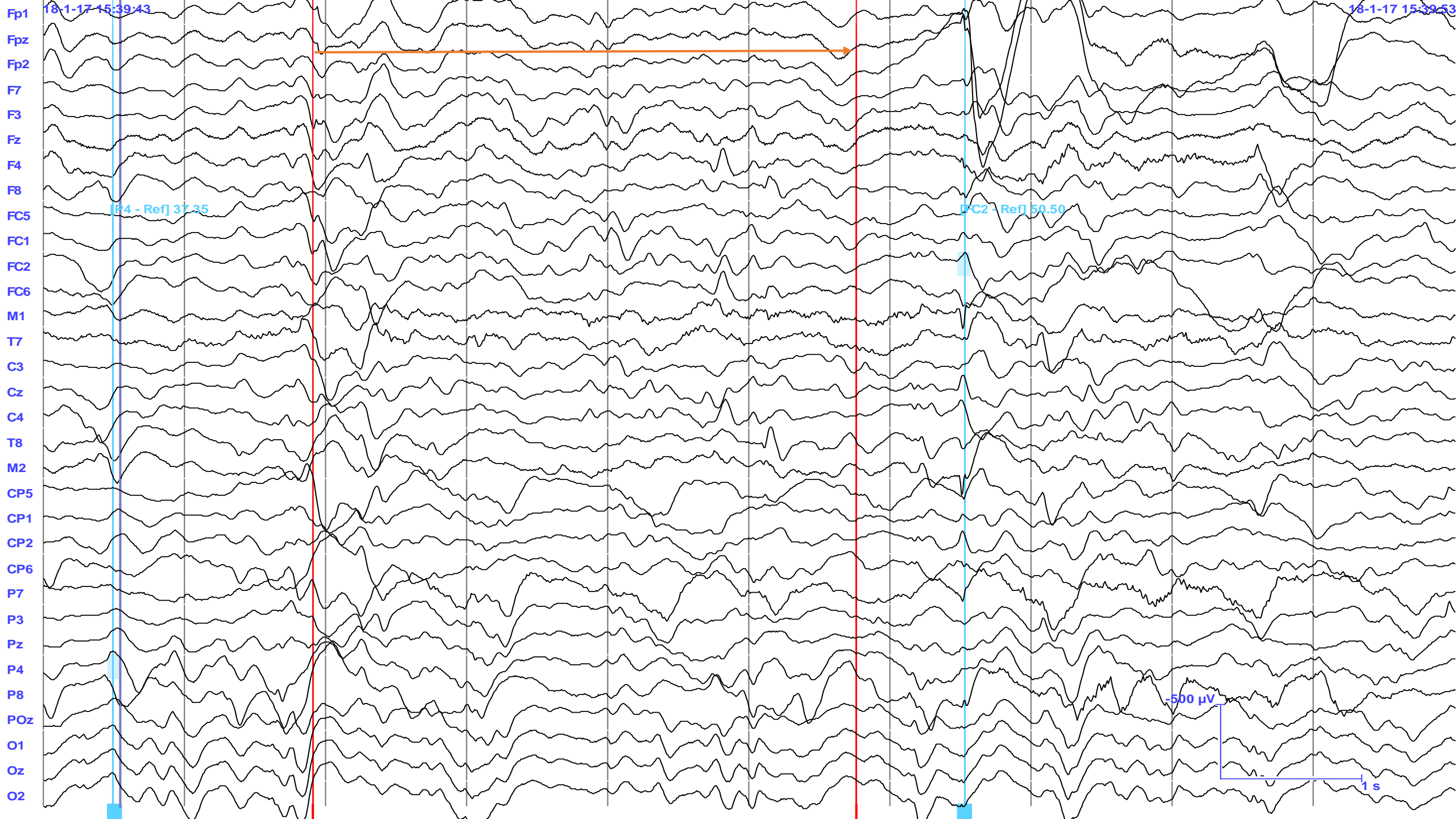

Supplement: FIGURE S1 — Raw EEG activity during tonic-myoclonic seizure. Red vertical lines represent the onset and offset of the seizure, orange horizontal line represents the duration of the seizure (close to 4 seconds). The left purple line represent interictal epileptic discharges on several channels (e.g., P8, C4, CP2) at approximately one second before the seizure onset. The light-blue vertical lines mark specific channels that display IED onset before (at P4 electrode) and after (at FC2 electrode) the seizure. The entire figure represents 10 seconds of EEG, each second indicated by gray vertical lines. [file Data_Sheet_1.PDF]
